# Comparative docking analysis of tyrosine kinase inhibitors with HER2 and HER4 receptors

**DOI:** 10.6026/97320630018974

**Published:** 2022-10-31

**Authors:** Priyanka Sonar, Karimunnisa Shaikh, Sangeeta Ballav, Soumya Basu, Sunil Harer

**Affiliations:** 1 Department of Pharmaceutics, Progressive Education Society's, Modern College of Pharmacy, Nigdi, Pune, M.S, India; 2 Cancer and Translational Research Laboratory, Dr. D.Y. Patil Biotechnology and Bioinformatics Institute, Dr. D.Y. Patil Vidyapeeth, Tathawade, Pune, M.S, India; 3 Department of Pharmaceutical Chemistry, Dattakala Shikshan Sanstha’s, Dattakala College of Pharmacy, Pune, MS, India

**Keywords:** HER 2, HER 4, molecular docking, tyrosine kinase receptors

## Abstract

Tyrosine kinase receptors promote the growth and differentiation of normal breast and malignant human breast cancer cells, known as ERBB receptors. Various ERBB receptors are EGFR/ErbB1 and ErbB2/neu, which get over expressed in different solid tumors that activate upon binding of ligand to the extra cellular domain of these receptors. Of note, the epidermal growth factor receptor (EGFR) is a prime contributor to cancer through the involvement of four receptor tyrosine kinases (RTKs), namely, HER1, HER2, HER3, and HER4. Among them, HER2 and HER4 are majorly associated with breast cancer. Non-peptide quinazoline compounds homologous of the adenosine triphosphate (ATP) are competitively inhibited to RTKs to prevent cancer growth and metastasis. Various small drug molecule that targets the RTKs having the same scaffold, includes Lapatinib, Tivozanib, Erlotinib, Gefitinib, Crizotinib, and Ceritinib. The present study aims to investigate the comparative potential of structurally similar TKIs against HER2 and HER4 receptor receptors-silico molecular docking using FlexX software (LeadIT 2.3.2). Each docked complex's interaction profile was performed using BIOVIA Discovery Studio Visualizer 4.0. Molecular docking analysis was performed in order to get deeper insights into the interaction and binding pattern of the ligands with HER2 and HER4 receptors. The docking results revealed the Lapatinib compound acquired the relatively highest binding score of -32.36 kcal/mol and -35.76 kcal/mol with HER2 and HER4 proteins, respectively, concerning other compounds. Lapatinib is identified as a potential inhibitor for both the RTKs. Our study thus suggests the probable direction that could be further explored in inhibiting EGFR protein harboring breast cancer.

## Background:

The ERBB receptorsare tyrosine kinase family receptor that promotes growth and differentiation of both normal breast and malignant human breast cancer cells [1]. Epidermal growth factor receptor (EGFR/ErbB1), is one member of this family, over expressed in 20% to 80% of breast cancers [2, 3], and another member is HER2 (ErbB2/neu), is amplified and/or over expressed (i.e., HER2-positive) in 20% to 30% of breast cancers [4, 5]. EGFR and HER2 have emergedas promising targets for cancer therapy that drive tumor growth and progression. The EGFR family comprises four distinct membrane tyrosine kinase receptors; EGFR/ErbB-1, HER2/ErbB-2, HER3/ErbB-3 and HER4/ErbB-4, which are activated upon ligand binding to the extracellular domain of these receptors [6-7]. Formation of receptor homo- or hetero-dimers resulting in phosphorylation of tyrosine kinase residues and cross-phosphorylation, that triggers numerous signaling pathways such as phosphatidylinositol-3 kinase (PI3K), mitogen-activated protein kinase/extracellular signal-regulated kinases (MAPK/ERK1/2), signal transducer and activator of transcription (STAT), phospholipase C (PLCγ), and/or the modulation of calcium channels [8], This sequence of events induces cellular responses which include proliferation, differentiation, and inhibition,of apoptosis, giving rise to diseases such as cancer [9]. The Tyrosine kinases are non-peptide aniline quinazoline compounds homologous of the adenosine triphosphate (ATP). This similarity allows them to compete for the ATP-binding domain of protein kinases preventing phosphorylation and subsequent activation of the signal transduction pathways, leading to apoptosis and decreasing cellular proliferation [10]. Under physiological conditions, the intrinsic activities of receptor tyrosine kinase inhibitors (RTKs) are strictly controlled [11]. Over expressed or increased activities of RTKs resulting from mutations, gene rearrangement or amplification have been correlated with tumor development and progression [12]. The epidermal growth factor receptor (EGFR), the first identified receptor of tyrosine kinases, is important for epithelial cell biology. It has been reported that EGFR is over expressed in various solid tumors such as gastrointestinal tract, non-small cell lung, breast, prostate, bladder, and ovarian carcinomas, head and neck cancers, and glioblastoma [13]. In the last decades, since the understanding of the key roles of RTKs in tumor development and progression, inhibition of RTK to prevent cancer growth and metastasis has become an attractive approach for the discovery of novel anticancer drugs [14]. The FDA has approved several small molecule receptor tyrosine kinase inhibitor drugs until September 2021, and additional inhibitors were approved by other regulatory agencies based on the various scaffold, that include Lapatinib, Tivozanib, Erlotinib, Gefitinib, Crizotinib, and Ceritinib [15]. Therefore, it is of interest to investigate the comparative potential of structurally similar TKIs against HER2 and HER4 receptors using molecular docking studies.

## Materials and Methods:

### Ligand and receptor preparation:

The SDF filesfor the drugs, Lapatinib (CID:208908), Erlotinib (CID: 176870), Gefitinib (CID:123631), Tivozanib (CID:9911830), Crizotinib (CID:11626560) and Ceritinib (CID:57379345) were downloaded from the PubChem database (https://pubchem.ncbi.nlm.nih.gov/) to serve as the docking ligands. These compounds were further prepared in Schrodinger Ligprepwizard. As Lapatinib is considered a potent inhibitor of HER2 and HER4 kinases, hence, it was used as a reference. The 3D X-ray crystal structure of two proteins; namely; HER2 kinase (ERBB2) (PDB: 3PP0) and HER4 kinase (ERBB4) (PDB: 3BBT) has resolutions of 2.25Å and 2.80 Å respectively were retrieved from Protein Data Bank (PDB)(https://www.rcsb.org/structure/2ZNN) [16, 17]. All of the bound ligands, water molecules, and other subunits were removed from both the proteins with the assignment of hydrogen atoms using the Protein Preparation Wizard (PPW) [18]. The positions of proteins and structure of ligands were minimized and optimized using the Optimized Potentials for Liquid Simulations-2005 (OPLS_2005) force field in Maestro [19]. Further, ionization states of the proteins were imparted by allotting amino acid chains using PROPKA program tool of PPW at pH 8.0.

### Molecular docking study:

A molecular docking study was performedin order to explore the structural features of the ligands with the proteins using FlexX software (LeadIT 2.3.2) [20]. The active site of the protein was defined by selecting the amino acid residues around the resolutions 2.25Å and2.80 Å of HER2 kinase (PDB: 3PP0) and HER4 kinase (PDB: 3BBT) respectively. The prepared ligands and proteins were fed into the docking protocol assigned with default parameters. The best conformation was determined by the binding affinity of ligands with the proteins. The interaction profile of each complex was performed by using BIOVIA Discovery Studio Visualizer 4.0.

## Results and Discussion:

Taking into note, epidermal growth factor receptor (EGFR) is a prime contributor of cancer through the involvement of four receptor tyrosine kinases (RTKs), namely, HER1, HER2, HER3, and HER4.

Among them, HER2 and HER4 are majorly associated with breast cancer, and identification of their potential inhibitors is highly desired. Therefore, we performed molecular docking analysis in order to gain deeper insights into their interaction and binding pattern with the ligands. The docking results revealed that the reference compound, Lapatinib acquired the highest scores of -32.36 kcal/mol and -35.76 kcal/mol with HER2 and HER4 proteins respectively over all other compounds (Table 1 - see PDF). Our study identified Lapatinib as a potential inhibitor for both the RTKs. As shown in Figure 1 (a)(see PDF), Lapatinib is able to establish six Hydrogen bonds (H-bonds) within the HER2 protein active site; the first one between quinazoline nitrogen and MET801 (1.88 Å), the second one between quinazoline carbon and GLN799 (2.51Å), third one between fluorine halogen atom and THR798 (2.68 Å), fourth one between a carbofluoro of flourophenyl and SER783 (2.84 Å), fifth one between methoxy carbon and ASP863 (2.19 Å) and sixth one between sulfonyl hydrogen and ASP808 (2.52Å).

In molecular docking interaction with HER4 receptor, Lapatinib was seen to secure eight H-bonds; two between the fluorine halogen atom and THR771 (2.58 Å, 2.05 Å), the next two between the sulfonyl oxygen atom and CYS778 (2.95 Å, 3.09 Å) third one between quinazoline nitrogen and MET774 (2.17Å), one between the sulfonyl oxygen atom and GLY777 (2.57 Å), other one methoxy carbon and ASP836 (Å), next interaction between the oxygen atom of Furan and GLU781 (2.42Å), last one between quinazoline carbon and GLN772 (4.30 Å) (Figure 2 -see PDF). Comparatively, the other ligands did not exhibit good docking scores with the proteins while Erlotinib rendered the least affinity (Table 2 - see PDF). Besides, Ceritinib was found to be incompatible with the HER2 receptor and did not bind to it. Thus, the binding score order for HER2 protein was Lapatinib> Crizotinib> Gefitinib> Tivozanib> Erlotinib whereas for HER4 was Lapatinib > Tivozanib > Crizotinib > Gefitinib > Ceritinib > Erlotinib.

## Conclusions:

Comparative docking study output of various tyrosine kinase inhibitors with Human epidermal growth factor receptor 2 (HER2) and HER2 suggest the promising binding response of Lapatinib over the other ligands. This study proposed that these drugs had represented the HER2 and HER4 receptors as targets of the epidermal growth factor receptor family. It is our aim to create increasing interest in utilizing these drugs in combination with chemotherapy and /or other HER2-directed agents in patients with central nervous system involvement, TKIs have shown to be effective in this setting for which treatment options have been previously limited and the prognosis remains poor. The aim of this study is to summarize the potential molecular docking interactions of currently approved TKIs for HER2+ breast cancer, and in non-small cell lung cancer supporting their use in key clinical trials, and in current clinical practice.
